# *Tuber wenchuanense*, a holarctic truffle with a wide range of host plants and description of its ectomycorrhiza with spruce

**DOI:** 10.1007/s00572-022-01097-y

**Published:** 2023-01-13

**Authors:** Piotr Mleczko, Dorota Hilszczańska, Filip Karpowicz, Maciej Kozak, Marco Leonardi, Aleksandra Rosa-Gruszecka, Anna Tereba, Giovanni Pacioni

**Affiliations:** 1 Institute of Botany, Faculty of Biology, Jagiellonian University, Gronostajowa 3, 30-387 Kraków, Poland; 2 Department of Forest Ecology, Forest Research Institute, Sękocin Stary, Braci Leśnej 3, 05-090 Raszyn, Poland; 3 Kraków, Poland; 4 Department of Life, Health and Environmental Sciences, University of L’Aquila, 67100 L’Aquila, Italy

**Keywords:** Rufum clade, Hypogeous fungi, *Tuber* ectomycorrhiza, Ascomycetes

## Abstract

*Tuber wenchuanense* ascomata (Ascomycota, Pezizales), a species originally described from Sichuan (China), were found in the Tatra Mountains in southern Poland. The purpose of this work was to (i) report and assess the first case of the holarctic natural distribution of a *Tuber* species, (ii) amend the original description of the species, (iii) summarize data on its host plants and (iv) describe its ectomycorrhiza. Specimens of *Tuber wenchuanense* from the Tatra Mountains were studied morphologically and molecularly. The ectomycorrhiza of this truffle with *Picea abies* was described for the first time. The distribution of *T. wenchuanense*, which is reconstructed based on sequences deposited in the publicly available nucleotide sequence databases, makes it the first holarctic *Tuber* species and the one with the northernmost habitat. In fact, its habitat is confined mainly to mountain coniferous forests and alpine and arctic tundra; although, according to known observations, the fruiting bodies of *T. wenchuanense* can be produced only under conifers. Based on the sequences of the internal transcribed spacer, this species appears to have low genetic variability over the entire distribution range. The phylogenetic tree showed that some of the unidentified phylotypes from the Rufum clade found by other researchers belong to *T. wenchuanense*. The ecological implications of these findings are discussed.

**Supplementary Information:**

The online version contains supplementary material available at 10.1007/s00572-022-01097-y.

## Introduction

The species of the genus *Tuber* (Ascomycota, Pezizales, Tuberaceae) establish different symbioses (mycorrhiza and endophytism) with the roots of several tree and shrub species (Pacioni and Comandini 1999), as well as with orchids (Selosse et al. 2004; Schiebold et al. 2018) and grasses (Schneider-Mauroury et al*.* 2020; Taschen et al. 2020). Mainly by virtue of the mutualistic relationship with trees, these fungi produce subterranean (hypogeous) fruiting bodies (ascomata) known as truffles. Truffles produce spores sequestered within the surrounding tissues, which are released and dispersed by animal consumption, similar to that of all underground mushrooms (Trappe et al. 2009; Ori et al. 2018; Urban 2016; Zambonelli et al. 2017). Most *Tuber* species prefer well-drained soils with high contents of calcium. The conditions to produce fruiting bodies are stricter than those where mycelia and mycorrhizae form, as has been well studied for cultivated species (Le Tacon et al. 2014; Pacioni et al. 2014).

Due to their strong dependence on symbiotic plants, on edaphic conditions, and on zoochory, the native range of the various species of *Tuber* seems to cover rather limited areas (Stobbe et al. 2013; Zambonelli et al. 2017; Ori et al. 2021). For a long time, knowledge of the distribution of *Tuber* species was based on the discovery of their ascomata. However, the number and reliability of records were limited because of both the hypogeous habit of fruit bodies and problems with the correct species delimitation and identification caused by the small number of morphological characteristics, mainly peridium and spores.

Thanks to the arrival of molecular tools, we are now witnessing a rapid increase in the number of new species being described, especially in previously neglected geographic areas, e.g. China and Northern-Central America. Additionally, the number of species records increases due to the molecular identification of their ectomycorrhiza and the mycelium present in the soil (Leonardi et al. 2021a, b). Bonito et al. (2010) subjected the *Tuber* sequences, which originated from both the ascomata and ectomycorrhizae/soil mycelia, present in public databases to phylogenetic analysis, obtaining a phylogenetic tree that revealed the existence of numerous undescribed species. After this pivotal paper, the number of *Tuber* species has grown very rapidly because of the application of molecular tools to taxonomy using predominantly ITS nrDNA as a barcode region.

The analyses of the ITS sequences of the reference taxa made by Bonito et al. (2010, 2013) and Bonito and Smith (2016) show that *Tuber* species are distributed rather regionally and seem to be naturally not shared between continents, if they are not mediated by human activity, such as the introduction of alien plants in gardens, parks, reforestations or orchards (Vellinga et al. 2009; Bonito et al. 2010). Cases where individual *Tuber* phylotypes (ITS similarity > 99%) are found on multiple continents, such as *T. aestivum*, *T. melanosporum*, *T. indicum*, *Tuber* sp. 19 and *Tuber* sp. 57 (Bonito et al. 2010), appear to be examples of recent human-mediated introductions. Thus, leaving aside past reports not genetically verified, the publicly available nucleotide sequence databases are now a reliable source of reference data for the geographical distribution analyses of the *Tuber* species.

The purpose of this work is to (i) report and assess the first case of a natural distribution in the three continents of the northern hemisphere, excluding tropical regions (Holarctic), of a *Tuber* species, namely, *Tuber wenchuanense* L. Fan & J.Z. Cao, based on our own data and the sequences retrieved from the publicly available nucleotide sequence databases, (ii) amend the original description of the species, (iii) summarise data on its host plants and (iv) describe its ectomycorrhiza with *Picea abies* (L.) H. Karst.

The inspiration for undertaking this research was the recent discovery of the ascomata of *Tuber wenchuanense,* a truffle belonging to the Rufum clade, originally described by Fan et al. (2013) from China, in the Tatra Mountains in southern Poland, for the first time outside the location of its protologue.

## Materials and methods

### Specimens

Ascocarps of *Tuber wenchuanense* were found during fieldwork carried out in the Polish part of the Tatra Mountains, Western Carpathians, in 2019 and 2020, without the aid of a trained dog. The geographical coordinates of localities were obtained using the Garmin 62 s GPS tool. The geographic coordinates of the study area are not given here for reasons of site protection, but they can be provided upon reasonable request. In 2020, the vegetation in the areas of ascocarp presence was characterised, presumptive host plants were identified and notes were made on the morphological characteristics and the odour of fresh ascocarps. In the same year, two soil samples (p. 10 × 10 × 10 cm) were excavated from the vicinity of ascocarps of the collection KRA F-TPN/20/0004 to obtain ectomycorrhizae. The vouchers of collections are preserved in the Jagiellonian University in Krakow (KRA) and the University of L’Aquila (AQUI) herbaria. For details on the specimens examined, see our description of *T. wenchuanense* in Supplementary material (SM. 1).

### Microscopy

#### Ascoma

The microscopic characteristics of spores, asci and peridium were examined on hand-made sections or squash preparations obtained from dried specimens. Each sample was rehydrated for 10 min in 20% KOH, rinsed with sterile water and then soaked with 3% KOH following the procedure described by Leonardi et al. (2019). Observations and measurements were made under a Zeiss AXIO imager2 microscope, and images were captured by a Leica DFC320 camera. Only fully mature spores in which the episporium was clearly distinguishable were considered for the analyses. For scanning electron microscope (SEM) observations, air-dried spores were covered with gold and analysed using the Hitachi S-4700 microscope (Laboratory of Scanning Electron Microscopy and Microanalysis, Institute of Geological Sciences, Jagiellonian University in Kraków).

The colours were determined using the mycological colour chart of Rayner (1970) (R) at 400 × magnification with a 5000 K light source without a filter. The measurements of microscopic characteristics were carried out at 400 × or 1000 × magnification for fine details.

#### Ectomycorrhiza

The roots from soil samples were carefully washed out in tap water, and ectomycorrhizae were isolated under a dissecting microscope and divided into morphotypes (Agerer 1991, 2006). The only morphotype in the samples possessing the characteristic indicative of a possible *Tuber* affinity, that was also relatively frequent in the samples, was chosen for further molecular identification and detailed macro- and micromorphological characterization. Ectomycorrhizae were placed in water and photographed using a Nikon DS M5 digital camera mounted on a Nikon SMZ-U dissecting microscope and coupled with a LUCIA ver. 4.82 image acquisition and analysis program. Representative ectomycorrhizal tips of the selected morphotypee were preserved in (i) CTAB buffer (for molecular identification) and (ii) FAA preservative solution.

Morphological characterisation followed the methods of Agerer (1991, 2006, 2004–2013). The ectomycorrhizae preserved in FAA were rehydrated in distilled water, and mantle scrapings were peeled off using a dissecting needle and mounted in concentrated lactic acid. Ectomycorrhizal cross and longitudinal sections were also made and mounted in lactic acid. Preparations were observed using a ZEISS AxioScope A1 microscope with a DIC system. The photos were obtained using a digital camera ZEISS AxioCam MRc5 coupled with the image acquisition and analysis program ZEN ver. 2012 blue edition. Quantitative values of microscopic characteristics were based on at least 30 measurements and are given as follows: (extreme minimum) mean (extreme maximum).

### Molecular analysis

#### DNA isolation

Genomic DNA from both fungal and plant tissues was extracted using a DNA isolation kit (NucleoSpin Plant II, Macherey–Nagel, Düren, Germany) according to the manufacturer’s protocol. Final DNA elution was performed in a volume of 100 µl elution buffer. The quality of the isolated material was determined by a UV–VIS NanoDrop One spectrophotometer or by 1% agarose gel electrophoresis. The obtained isolates were stored at − 20 °C until further analysis steps.

#### Amplification of fungal DNA

For optimization of target ITS nrDNA fragment amplification, two primer combinations were used: (1) ITS1 (5′- TCC GTA GGT GAA CCT TGC GG -3′) and ITS4 (5′- TCC TCC GCT TAT TGA TAT GC -3′) (White et al. 1990) and (2) ITS1f (5′-CTT GGT CAT TTA GAG GAA GTA A-3′) (Gardes and Bruns 1993) and ITS4. For each PCR (polymerase chain reaction) run performed, a negative control (control of reagent contamination) and a positive control with 100 ng of high-quality genomic DNA of *Hymenogaster* sp. (quality control of reagents and reaction settings) were always additionally made. The PCR was run under the following conditions: initial denaturation 95 °C for 15 min, denaturation 95 °C for 20 s, primer annealing 55 °C for 40 s, elongation 72 °C for 1 min and final elongation 72 °C for 10 min. The number of cycles for the reaction was 35. Each PCR was performed in a volume of 20 µl, with the following composition: 4 µl Gold Hot Start PCR Mix Load (Syngen Biotech, Wrocław, Poland), 0.5 µl (10 µM) of each primer, 13 µl of water, PCR grade and 2 µl of extracted DNA. Amplification products were visualized electrophoretically on a 1.5% agarose gel with the addition of a Green DNA Gel Stain (Syngen Biotech, Wrocław, Poland). The PCR products with successful amplification of the genetic marker (lack of non-specific products) were purified using Clean up Kits (A&A Biotechnology, Gdańsk, Poland). Attachment of the labelled terminator was then performed using the BigDye® Terminator v3.1 Cycle Sequencing Kit (Thermo Fisher Scientific, Waltham, Massachusetts, USA). The reaction was prepared in a total volume of 10 µl at the following ratios: 2 µl reaction buffer, 2 µl BigDye® Terminator Mix, 2.9 µl water, 1.6 µl starter (10 µM) and 1.5 µl purified PCR product. The reaction conditions were as follows: initial denaturation 96 °C for 1 min, denaturation 96 °C for 10 s, primer annealing 55 °C for 5 s and elongation 60 °C for 4 min. Twenty-five reaction cycles were run. Purification of the reaction products from free terminators and salts was performed using the BigDye XTerminator® Purification Kit (Thermo Fisher Scientific, Waltham, Massachusetts, USA) in a mixture of 55 µl of the SAM solution (45 µl) and XTerminator (10 µl). The resulting reaction products were sequenced on an ABI 3500 capillary sequencer (Applied Biosystems, Waltham, Massachusetts, USA).

#### Amplification of host plant DNA

Fragments of the cpDNA genes transfer RNA (trnL) and ribulose-1,5-bisphosphate carboxylase/oxygenase large subunit (rbcL) sequence analyses were used to identify the taxonomic affiliation of the ectomycorrhizal plant symbiont. Amplification of the studied regions was performed using primers c (5′ -CGA AAT CGG TAG ACG CTA CG- 3′) and f (5′- ATT TGA ACT GGT GAC ACG AG-3′) for trnL and 1F (5′- ATG TCA CCA CAA CTA ACA GAA AC-3′) and 1352R (5′- CAG CAA CTA GTT CAG GRC TCC-3′) for rbcL (Taberlet et al. 1991; Manen et al. 2004). The PCR conditions were as follows: initial denaturation 94 °C for 5 min, then 35 cycles, denaturation 92 °C for 45 s, primer attachment 57 °C for 45 s, extension 72 °C for 2 min and final extension 72 °C for 10 min. The reaction was performed in a volume of 25 µl in a mixture whose composition was identical to that used in the amplification of the fungal markers. Negative (2 µl of water) and positive controls (100 ng of high-quality genomic DNA of *Allium* sp.) were always present during the reaction. The success of the PCR was checked by separating the products on a 1% agarose gel. The gel bands were isolated from many nonspecific products. The gel bands and PCR products were purified using the GeneMATRIX Basic DNA Purification Kit (EurX, Gdańsk, Poland) according to the manufacturer’s protocol. Sequencing using the Sanger method (Sanger et al. 1977) was contracted to an outside company with primers used to amplify the trnL gene fragment and primer 1F used to amplify the rbcL gene fragment.

New sequences were submitted to GenBank. Their accession numbers are reported in Table 1, along with other sequences analysed in this study.

**Table 1:** Accession numbers (GenBank (Sayers et al. 2021) and UNITE (Kõljalg et al. 2020) in italics) and collection localities of the specimens analysed in this study. If in the Polish collection, the herbarium voucher for the ectomycorrhiza and the ascoma specimen are identical, the ectomycorrhiza voucher is marked with an asterisk (*)

Accession no	Name labelled	Species annotation	Origin	Location	Host	Reference
AY748863	Uncultured ECM (*Tuber*)	isolate BYD8a	ECM	Poland	*Salix capreae*	GenBank, K. Hrynkiewicz, pers. comm
EF362475	*T. rufum*		Asc	Italy	unknown	Iotti et al. 2007
JX267044	*T. wenchuanense*	HMAS 60,239, holotype	Asc	China, Sichuan	*Larix mastersiana*	Fan et al. 2013
JX630357	Uncultured *Tuber*	HI_S3_6_21_1	ECM	USA, Alaska	*Salix arctica*	Timling et al. 2012
JX630932	Uncultured *Tuber*	AR3	ECM	Canada, Bank Island	*Salix arctica*	Timling et al. 2012
KF617424	Uncultured fungus	3242O15	ECM	USA, Alaska	*Picea mariana*	Taylor et al. 2014
MK211278	*T. pustulatum*	AQUI 9725	Asc	Spain	*Corylus avellana*	Leonardi et al. 2019
MK211283	*T. theleascum*	ITVC908 holotypus	Asc	Mexico	*Quercus* spp., *Arbutus* sp.	Leonardi et al. 2019
MK211284	*T. theleascum*	ITVC 957	Asc	Mexico	*Quercus* spp.	Leonardi et al. 2019
MW632951	*T. zambonelliae*	MUB_Fung-0995 holotypus	Asc	Spain	*Quercus ilex* subsp. *ballota*	Crous et al. 2021
MW632953	*T. zambonelliae*	MUB_Fung-0741	Asc	Spain	*Quercus ilex* subsp. *ballota*	Crous et al. 2021
MZ423173	*T. aestivum*	AQUI 10,150 epitype	Asc	Italy	*Tilia cordata*	Leonardi et al. 2021a; b
ON704774	*T. wenchuanense*	TPN/19/0293	Asc	Poland	*Picea abies*	This study
ON704775	*T. wenchuanense*	TPN/20/0002	Asc	Poland	*Picea abies*	This study
ON704776	*T. wenchuanense*	TPN/19/0298	Asc	Poland	*Picea abies*	This study
ON704777	*T. wenchuanense*	TPN/20/0001*	ECM	Poland	*Picea abies*	This study
ON704778	*T. wenchuanense*	TPN/20/0001	Asc	Poland	*Picea abies*	This study
ON704779	*T. wenchuanense*	TPN/20/0004	Asc	Poland	*Picea abies*	This study
ON704780	*T. wenchuanense*	TPN/20/0004*	ECM	Poland	*Picea abies*	This study
UDB0221220	*T. wenchuanense*	m54032_171018_101447/33096216/ccs	Soil	Estonia	*Picea abies* predominant	Tedersoo et al. (2014)
UDB0452874	*T. wenchuanense*	EST14263696	Soil	Estonia	*Picea abies* predominant	Tedersoo et al. (2014)
UDB0690980	*T. wenchuanense*	EST16_292144	Soil	Estonia	*Picea abies* predominant	Tedersoo et al. (2014)
Further reports without sequences released						
SH210422.07FU	*T. wenchuanense*	VRSIC_01, 02	ECM	Slovenia	*Bistorta vivipara*	Arraiano-Castilho et al. (2021), personal comm
SH210422.07FU	*T. wenchuanense*	KRVAVE_01	Soil	Slovenia	*Dryas octopetala, B. vivipara*	Arraiano-Castilho et al. (2021), personal comm
SH210422.07FU	*T. wenchuanense*	LAUTAR_04	Soil	France	*D. octopetala, B. vivipara*	Arraiano-Castilho et al. (2021), personal comm
SH210422.07FU	*T. wenchuanense*	LAUTAR_05	Soil	France	*Salix herbacea, B. vivipara*	Arraiano-Castilho et al. (2021), personal comm

#### Bioinformatic processing of the sequences and phylogenetic analysis

The following programmes were used to visualise the chromatograms and to process and trim the sequences: AliViev v. 1.2.6 (Larsson 2014), BioEdit v. 7.2.5 (Hall 1999) and Geneious Prime v. 2020.2.4 (https://www.geneious.com). The taxonomic affiliation of the obtained sequences was determined by comparison with *T. wenchuanense* sequences from the NCBI (Sayers et al. 2021) and UNITE (Kõljalg et al. 2020) databases using the BLAST algorithm (Altschul et al. 1990). After excluding the ambiguous regions at the 5′ and 3′ ends of the chromatograms, the sequences were processed and aligned with the MAFFT programme (https://mafft.cbrc.jp/alignment/ software/) using the E- INS -i alignment strategy (Katoh and Standley 2013). This analysis included 22 nucleotide sequences generated during this study as well as those obtained from NCBI and UNITE databases (Table 1). The final data set contained a total of 660 positions. Evolutionary analyses were performed in MEGAX (Kumar et al. 2018). The model with the lowest AICc value (Akaike Information Criterion, corrected; Nei and Kumar 2000) was selected to describe the best substitution pattern. Maximum likelihood analysis was based on 1000 bootstrap replicates using a Kimura 2-parameter model with a gamma distribution of rates among sites (Kimura 1980). *Tuber aestivum* (MZ423173) was chosen as the outgroup taxon.

### Terminology

The description of the morphological characteristics of ascomas and ectomycorrhizae is in accordance with the definitions of Ainsworth & Bisby’s Dictionary of the Fungi (Kirk et al. 2008).

## Results

The morphology and molecular analyses of the ascomata led to *Tuber wenchuanense* (Fan et al. 2013). The original description (Fan et al. 2013) is based on herbarium specimens from a single collection from 1989 divided into holotype and isotype and is quite synthetic and not always clear. With respect to this, we provide a more complete description (Supplementary Material: SM. 1) which confirms, more clearly, that the ornamentation of the spores is first constituted by spines free at their bases which then become reticulated and with their tips fold into the hooks.

Without the ITS sequence, it would have been very difficult to morphologically identify this species basing solely on the description of the protologue in Fan et al. (2013). Molecular analysis of ectomycorrhizae pointed to *T. wenchuanense* as a fungal partner and identified *Picea abies* as the host plant, thus allowing ectomycorrhiza description.

### Phylogenetic analyses of T. wenchuanense sequences

**Fig. 1:**
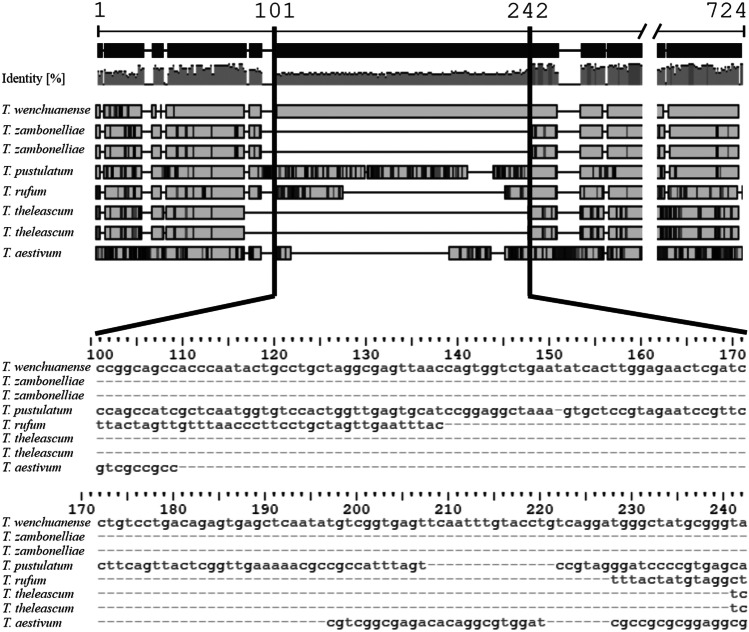
Schematic representation of supposed, 142-bp-long insertion in the hypervariable region ITS1 of *T. wenchuanense*. Identity means pairwise identity across all pairs in the column

All the sequences analysed by us, including AY748863 and JX630932 (both designated by Healy et al. (2016) as *Tuber* “sp. 58”), show a high similarity (above 98%) with the sequences of a type of *Tuber wenchuanense* L. Fan & J.Z. Cao. Comparison of all sequences of *T. wenchuanense* considered in this study with sequences of other *Tuber* taxa located in the NCBI and UNITE databases revealed a unique molecular pattern characteristic of this species in its ITS1 hypervariable region (Fig. 1). The observed pattern is a 142 bp fragment that could be the result of a large insertion. The fragment is unique among *T*. *wenchuanense* sequences and has very low similarity (65–69%) to other sequences available for comparison in the abovementioned databases. These characteristics of said insertion are making it a suitable, species-specific molecular marker, especially for high throughput sequencing-based studies.

We observed very low intraspecific variability (overall average *p*-distance = 0.0068) in ITS1-5.8S-ITS2 nrRNA region sequences in the geographically distant collections of *T. wenchuanense*. The total data set consisted of 22 sequences (including 15 sequences of *T. wenchuanense*, 6 sequences of closely related taxa from genus *Tuber* and the sequence MZ423173 of *Tuber aestivum*, which was used as an outgroup) and 724 aligned positions (ITS1 = 342, 5.8S = 149, ITS2 = 233), among which 354 were variable. The ML phylogenetic tree based on the ITS region (Fig. 3) recovered with good support for all the clades and species considered. Nodes with bootstrap values lower than 70% were eliminated. Specimens from Poland analysed in this study cluster in a clade together with all records showing very high ITS sequence similarities (> 99%) with the oriental species *Tuber wenchuanense* (Fan et al. 2013). Few sequences from the Tatra Mountains clustered together, forming a distinct subclade within the *T. wenchuanense* clade. This variation was caused by multiple site SNPs (single nucleotide polymorphisms, *n* = 3) occurring in all sequences forming this group.

### Ectomycorrhiza identification

**Fig. 2:**
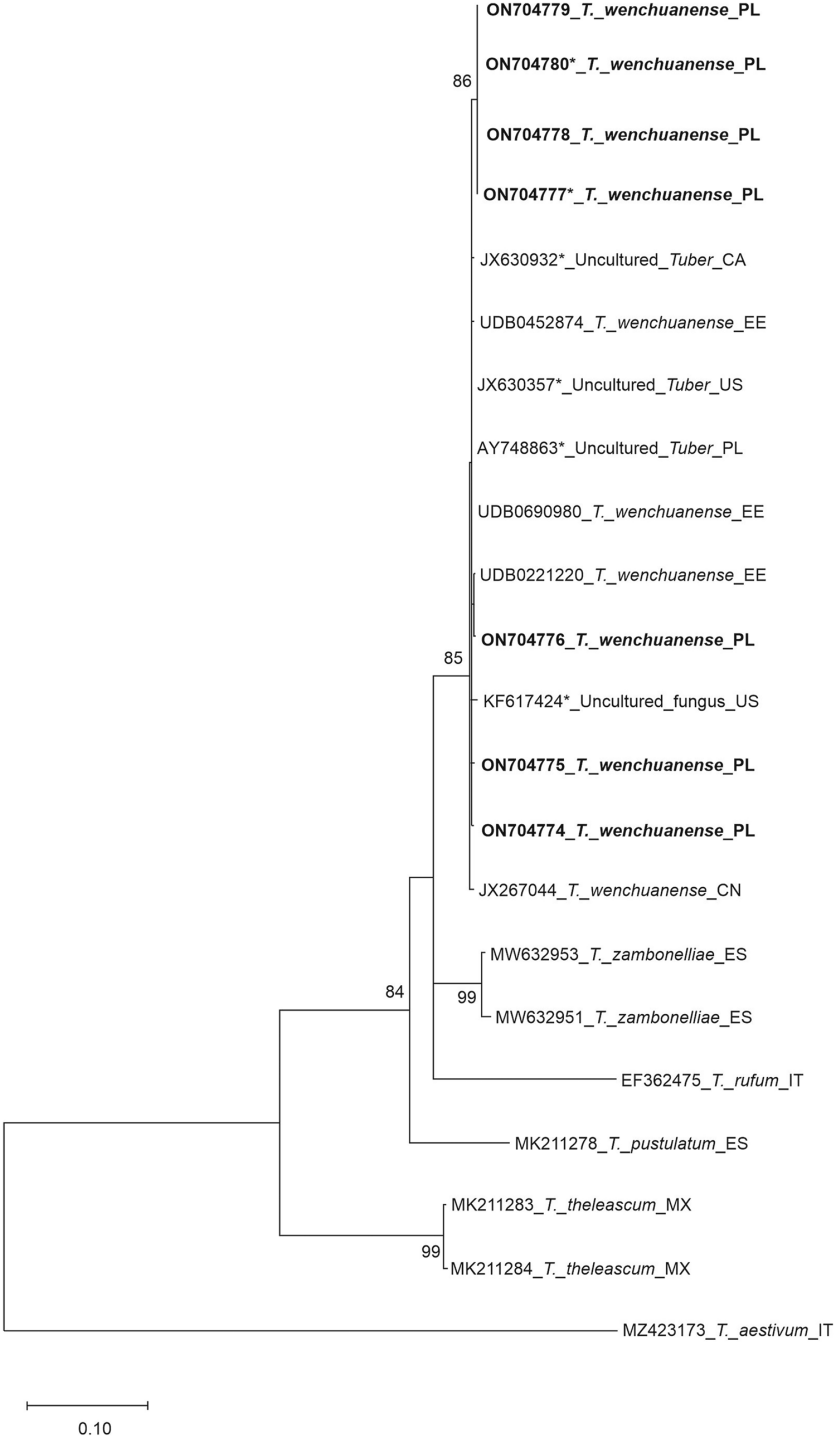
Maximum likelihood tree obtained from the alignment of ITS nuclear rDNA region sequences. Maximum likelihood phylogenetic analysis was inferred from the ITS nrDNA sequences of *Tuber wenchuanense* retrieved from GenBank and UNITE and included in Table 1. The tree displays the relationships of ascomata and ectomycorrhizae (*) of *T. wenchuanense* associated with *Picea abies*. Sequences obtained during this study are indicated in bold. Bootstrap values ≥ 70% are indicated on the nodes of branches. The tree is drawn to scale, with branch length measured from the number of substitutions per site. *Tuber aestivum* was included as an outgroup

Molecular analyses confirmed the match between the ascocarps and ectomycorrhizae collected in their vicinity (Fig. 2). The two sequences obtained for *rbcL* (ON814582) and *trnL* (ON814579) have affinities of 100% and 99.88%, respectively, with various species of *Picea*; however, only *Picea abies* was present in the sampling area. Thus, we conclude that this tree species was the host plant of *T. wenchuanense* ectomycorrhizae in the Tatra Mts.

### Description of ectomycorrhiza with Picea abies

Mycorrhizal system (Fig. S-6f) monopodial, up to 8 mm long, main axis app. 0.3–0.5 mm in diameter, side branches 0.3–0.4 mm in diameter, both main axis and side branches straight, rarely bent; mycorrhizal surface smooth or, on some side branches, covered with small scales coloured similarly to the mycorrhiza, cortical cells not shining through, emanating hyphae very sparse; young and actively growing tip cream-coloured, mycorrhiza beneath the tip yellow–brown to dull orange–brown in mature part, with a slightly reddish hue in the main axis (Fig. S-2).

**Fig. 3:**
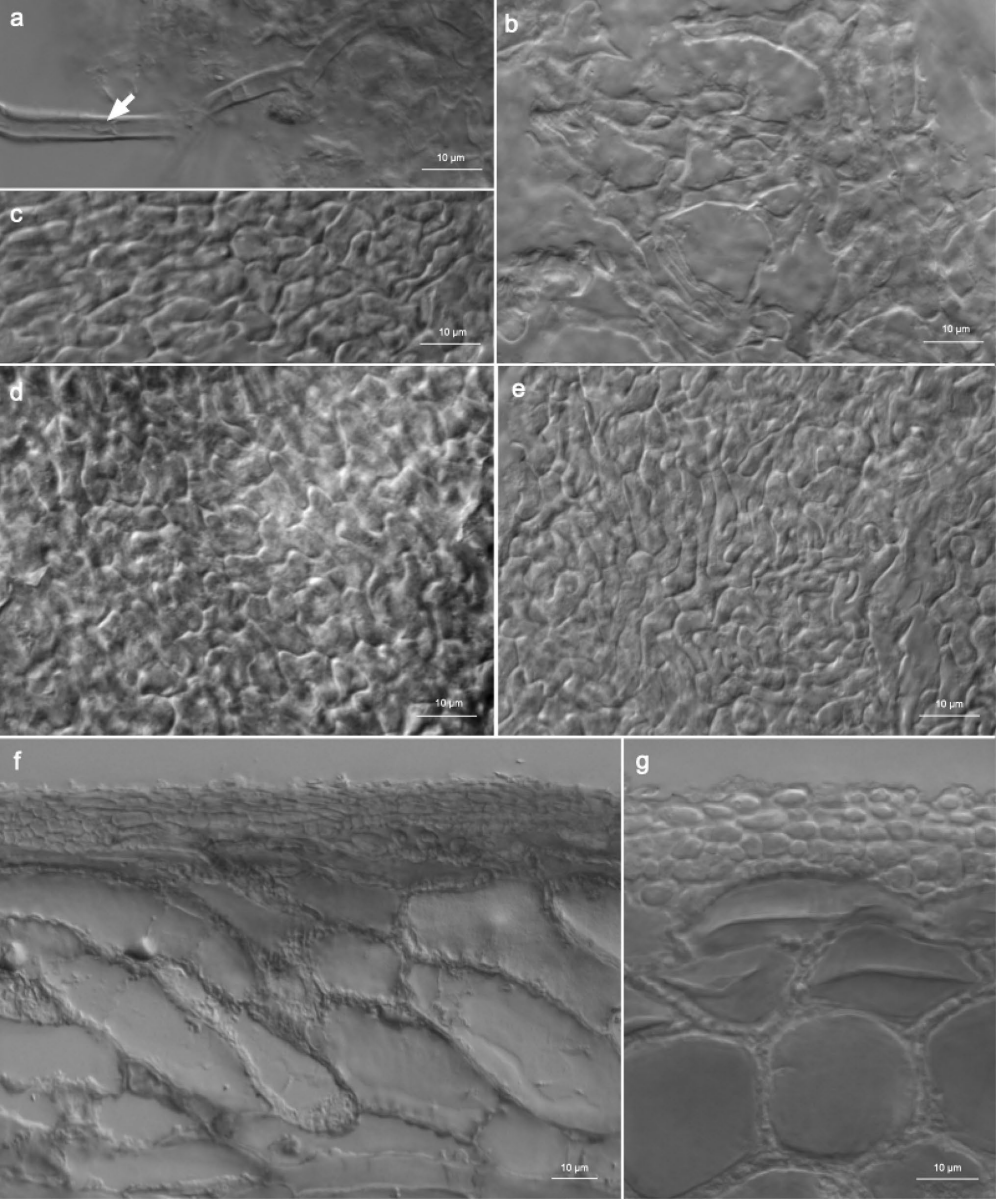
*Tuber wenchuanense* ectomycorrhizae microscopic structures: **a** emanating hypha; note intrahyphal hypha (arrow); **b** outer mantle surface—a hyphal net; **c**, **d** middle mantle layers—a structural variation; note transitional epidermoid cells in d; **e** inner mantle surface; **f**, **g** longitudinal **f **and cross **g **sections of the ectomycorrhizal tip. Scale bar: 10 μm (**a**–**g**) (herbarium voucher TPN/20/0001-ECM)

Mantle (Fig. 3b-e) of type Q/P (epidermoid/angular cells bearing a hyphal net). Outer mantle surface a hyphal net, hyphae branched and septated, cells (6.3) 14.5 (25.5) μm long and (2.4) 4.8 (8.7) μm in diameter, cell walls 0.4–5.7 μm thick, yellowish, septa thinner than the walls, hyphal surface smooth when thin-walled or cracked in the case of thick wall deposition. Hyphal cells beneath the net become gradually more irregular in shape and epidermoid, with intracellular spaces filled with matrix material (Fig. S-3). Middle mantle layers pseudoparenchymatous to irregularly plectenchymatous, hyphae densely arranged, hyphal cells of different shape, epidermoid, irregularly polygonal or elongated and branched, septated, hyphal cells (2.9) 12.9 (34.7) × (2.4) 6.4 (14.8) μm, walls yellowish, ca. 0.–1.0 μm thick, septa, when discernible, mostly thinner than the walls. Hyphal cells of the middle mantle in the tip region are similar in shape but of slightly smaller dimensions (Fig. S-4). Inner mantle surface plectenchymatous to pseudoparenchymatous, hyphae branched, undulating, (1.6) 2.7 (4.5) μm in diameter, thin walled (Fig. S-4).

Emanating hyphae (Fig. 3a) sparse, branched, app. 4 μm in diameter, walls smooth, yellowish-brown 0.6–0.7 μm thick, intrahyphal thin-walled hyaline hyphae present.

**Fig. 4:**
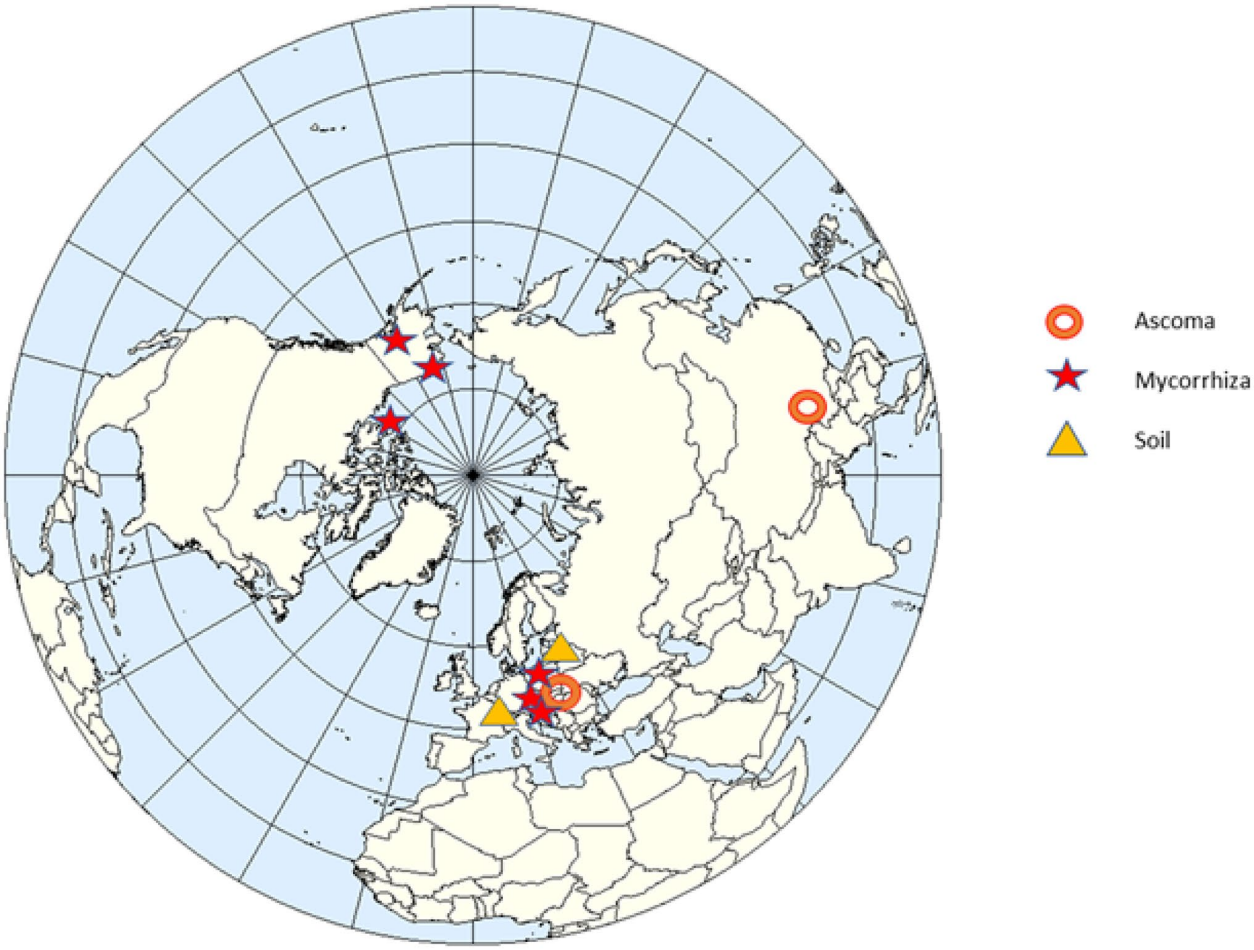
Hitherto known distribution of *Tuber wenchuanense*

Mycorrhiza in section (Figs. 3f, g, S-5). Mantle in cross Sect. 13.5–20.0 μm thick, three-layered, but the layers are rather discrete, clearly pseudoparenchymatous in all layers. Outer layer is built by one row of sparsely arranged hyphae, hyphal cells (3.23) 5.8 (10.9) × (2.2) 2.9 (5.4) μm, thick-walled; middle layer consisting of 1–3 rows of thick-walled, densely arranged hyphae, hyphal cells (3.4) 6.6 (13.5) × (2.1) 3.6 (6.3) μm; inner layer built by 1 row of thin-walled hyphae, hyphal cells (1.9) 5.5 (12.1) × (2.0) 3.8 (7.4) μm. Mantle in the longitudinal section is similar in structure and cell dimensions to this in the cross section. Tannin cells in the longitudinal section (Fig. 3f) are more or less flattened, positioned parallel to the long axis of ectomycorrhiza or somewhat diagonally, (27.2) 49.9 (85.7) μm long, (8.8) 13.2 (22.0) μm wide. Cortical cells in the longitudinal section (Fig. 3f) flattened in a majority, positioned alongside the long axis of ectomycorrhiza but diagonally, (21.8) 61.7 (108.9) μm long, (9.7) 21.5 (35.9) μm wide. Hartig net in section (Figs. 3f, g) (1.2) 3.2 (6.9) μm thick, built by 1 up to 3 rows of hyphae.

### Distribution and host range

*Tuber wenchuanense* exhibits a considerable geographic disjuncture (South-East China, Central Europe and Arctic America) with a general distribution in a Holarctic realm (Fig. 4). Its host plants comprise a wide range of species, both angiosperms and gymnosperms (Table 1): two species of spruce, *P. abies* (L.) H. Karst., *P. mariana* (Mill.) Britton, Sterns & Poggenb.; a larch species, *Larix mastersiana* Rehder & E.H. Wilson; three species of willows, *Salix caprea* L., *S. herbacea* L. and *S. arctica* Pall.; the mountain avens *Dryas octopetala* L. and a perennial herbaceous plant the alpine bistort *Bistorta vivipara* (L.) Delarbre (Arraiano-Castilho et al. 2021; pers. comm., Fan et al. 2013; Hrynkiewicz, pers. comm., Taylor et al. 2014; Tedersoo et al. 2014; Timling et al. 2012). In North America, the species was noted in the Arctic zone in Alaska and northwestern Canada. In Asia, it was found in Wenchuan Province located on the southeastern edge of the Qinghai-Tibet Plateau, which reaches altitudes up to 3000 m a.s.l. (the exact location of the protologue was not mentioned by Fan et al. 2013), and in all but one European locality, it was detected in cold environments: the subalpine coniferous forests and alpine vegetation of the Alps (Arraiano-Castilho et al. 2021) and the Tatra Mountains (this paper) and coniferous forests of the northern boreal zone (Tedersoo et al. 2014). The only European low-elevation locality was found in Poland in the southern Baltic Lake District, a subprovince of the North European Plain, which is under the influence of a mild continental and humid climate (K. Hrynkiewicz, pers. comm.).

Only the locality data of *T. wenchuanense* for Polish collections were obtained during the study. The geographical coordinates of the study area are not given here for reasons of site protection, as it is located in the Tatras NP. However, they can be provided upon reasonable request.

## Discussion

*Tuber* species in the Rufum clade are distributed across the Northern Hemisphere, with endemism centres in Europe, Asia and North America (Bonito et al. 2013). According to Bonito et al. (2010) and Healy et al. (2016), the Rufum clade is among the most diverse in the genus and includes numerous cryptic undescribed species. One of these species was “sp. 58”, designated by Healy et al. (2016) for sequences AY748863 and JX630932 deposited in the NCBI database, which currently must be identified as *T. wenchuanense* L. Fan & J.Z. Cao.

An unexpected and not sought-after result was that despite its disjoint wide distribution in the Holarctic, its occurrence in diverse habitats from the low elevations of Estonian forests (Tedersoo et al. 2014) and a coal mine of Poland (Hrynkiewicz, pers. comm.) to the alpine-arctic tundra, and a large repertoire of host plants, genetic analysis of *T*. *wenchuanense*, although restricted to the ITS1 region, shows surprising homogeneity within the group, supported by a low number of base differences per site of average evolutionary divergence between sequence pairs assessed using *p*-distance and Jukes-Cantor models.

The most likely explanation is that the various populations detected so far have been subject to continuous gene flow since recent times. Unfortunately, this argument can only be the subject of hypothetical speculations without the support of data that allow us to fully understand the ecological corridors between Europe and Asia and between Southeast Asia and North America as these are currently highly disjointed areas. However, a phenomenon similar to that observed in some tertiary plant relics in disjointed areas of Eurasia may also have occurred, in which non-coding genes, including ITS, show a slow pace of evolution, remaining rather conserved over time (Ballian et al. 2006; Fineschi et al. 2004).

*T. wenchuanense* appears to be a host-generalist fungus that therefore should have higher rates of establishment and spread because it has a greater likelihood of finding a suitable host (Vellinga et al. 2009). The ascomata of *T. wenchuanense* were known until now only from the locality of its protologue in China (Fan et al. 2013); to the best of our knowledge, the discovery made in the Tatra Mountains in Poland is a second currently known finding of the ascomata of this species. However, *T. wenchuanense* was identified previously by other researchers by analyses of ITS sequences obtained from ectomycorrhizae and from soil samples, both in Europe and North America (Timling et al. 2012; Taylor et al. 2014; Tedersoo et al. 2014; Arraiano-Castilho et al. 2021; Table 1). Considering all the available data, the distribution of this species can be described as Holarctic and covers all three continents: Europe, Asia and North America (Fig. S5).

Such a distribution is unique in that it is certainly natural, affecting only natural habitats and native plants. Previously, some *Tuber* species were known to occur in Europe and North America and were introduced to other continents by human activities (Vellinga et al. 2009). Apart from the obvious global spread of European *Tuber* species cultivated for their economic value, other cases of expansion of the original geographic range have been detected, basically with molecular methods. *Tuber rapaeodorum* Tul. & C. Tul. (current name *T*. *rapiodorum*), known in Europe under various deciduous trees, orchids and *Phragmites australis* (Poaceae), has also been introduced in North America and New Zealand. The human spreading of plants has unwittingly transferred many species of truffles, some of which are of economic interest, such as *T. floridianum*, following the Pecan orchards (Grupe et al. 2018). A very interesting case to investigate is the report of *T*. *magnatum* in a natural forest of Thailand, almost 9000 km from Italy, with ITS sequences identical to those of the Piedmontese type (Suwannarach et al. 2017).

*Tuber anniae* W. Colgan & Trappe is known in northwestern North America, the Baltics in Europe, and as an introduced species in New Zealand (Wang et al. 2013; Bulman et al. 2010). However, *T. anniae* is a species complex with three clades corresponding to disjunct populations (Wang et al. 2013), whereas in the case of *T*. *wenchuanense*, no cryptic species was detected based on ITS.

Mature specimens of *T. wenchuanense* can be characterized by the presence of two types of spores: spino-reticulated and free spiny with no connection ridges at spine bases (Fig. S-1). Spiny ornamentation is typically present in not fully ripe spores, where the spines are initially straight and reach up to 10 µm in length. Along with maturation, they fold into a hook at the apex, always remaining free at the base. The coexistence of spino-reticulated spores and spores with free spines at their bases was also found in other species of *Tuber,* such as those of the Indicum group (Chen et al. 2011; Kinoshita et al. 2018). The European diffusion of *T. wenchuanense* gives rise to the suspicion that in reality, it may have already been collected in Europe, perhaps with the name of *Tuber malacodermum* E. Fisch., a critical species of the Rufum clade with which it shares the pseudoparenchymatous peridium and spores with spines connected at their bases, whose type did not produce amplicons (Leonardi et al. 2019). The species with similar morphology are also *T. pustulatum* M. Leonardi, A. Paz, G. Guevara & Pacioni and *T. theleascum* M. Leonardi, A. Paz, G. Guevara & Pacioni, described recently by Leonardi and coworkers from Mediterranean Europe (continental Spain and Corsica, France) and Mexico, respectively (Leonardi et al. 2019). *Tuber wenchuanense* shares a similar two-layered peridium type, pseudoparenchymatous in the upper and plectenchymatous in the inner layers, with *T. theleascum*. However, the inner layer is much more pronounced in the latter species, and the spines of *T. wenchuanense* spores are connected at their bases by only a few ridges, which do not form a complete reticulum, a feature that is shared with *T. malacodermum* (Leonardi et al. 2019). Both *Tuber* species, *T. pustulatum* and *T. theleascum*, have reddish-brown to ferruginous peridium, whereas the colour of *T. wenchuanense* ascocarps is much paler (gray–brown to yellowish-brown) (Fan et al. 2013).

The ectomycorrhizae of *T. wenchuanense* share several features of the ectomycorrhizae of the other species from the Rufum clade, such as *T. rufum* and *T. huidongense*, i.e. a mantle that can be classified as epidermoid, with no cystidia and rather scarce emanating hyphae (Rauscher and Chevalier 1995; Rauscher et al. 1995; Wan et al. 2016). The typical feature of *T. rufum* ectomycorrhizae is a surface net formed by rather thick-walled hyphae. *Tuber wenchuanense* ectomycorrhizae are clearly similar to those formed by *T. rufum,* although some minor differences can be noted in the structure of the mantle (the middle layer tends to be a transitional type between epidermoid and irregularly polygonal in *T. wenchuanense* and epidermoid in *T. rufum*), mantle hyphae dimensions (slightly larger upper values of the middle mantle cells in *T. wenchuanense*: 14.8 and 34.7, versus 10.5 and 22.0 in *T. rufum*) and the thickness of the walls of the surface net hyphae (thicker in *T. wenchuanense*: up to 5.7, versus app. 3.0 in *T. rufum*). However, it can be concluded that the general morphology of the *T. wenchuanense* ectomycorrhizae resembles the one typical of *T. rufum,* which additionally supports the results of the sequence analyses and the investigation of morphological characteristics of ascomata.

The distribution range and the set of host plants of *T. wenchuanense* indicate a preference for cold and moist habitats such as mountain and boreal coniferous forests, alpine meadows and arctic tundra. Only one locality in northern Poland, known from ectomycorrhiza of *Salix caprea* growing in lowland habitat in a temperate climate, seems to elude this general pattern. From the data acquired thus far, it can be suggested that mountain coniferous forests are the environments most suitable for *T. wenchuanense* fructification, while in environments with more severe climates the species is present as mycorrhiza and mycelium only. However, in fact, most of our knowledge about the distribution of *T. wenchuanense* comes from the results of molecular investigations of ectomycorrhizae or soil samples and not the results of the search focused on hypogeous fruiting bodies. Hypogeous fungi are usually sought in forested areas, either by random digging in the ground or with the help of specially trained dogs. Arctic-alpine tundras are perhaps the least expected areas to search for these fungi. Recently, however, one of the co-authors (G.P.) received a *Hymenogaster* sp. found under *Salix herbacea* in the alpine meadow of the Rhodope Mountains (Bulgaria) and a black truffle (*Tuber* sp.) found near the Arctic Circle, both with the help of the trained truffle dogs. Thus, the use of these animals could open new perspectives for the diversity of hypogeous fungi in cold climates.

Plant species that we know to be associated with *T. wenchuanense* prefer cool or cold and humid climates, but the range of soils on which they occur is relatively wide, from poor podzol mountain soils characteristic of *L. mastersiana* forests (Shengxian 1995), organic soils preferred by *P. mariana* (Viereck et al. 1990), loose, fresh, moist clay soils preferred by *P. abies* (Alavi 1996), to the calcareous soils of *D. octopetala* (Harrington and Mitchell 2002). Despite these differences, all of these plants require well-drained and relatively moist soils. They also have a wide distribution range, e.g. the area occupied by the common spruce *P. abies* extends from the Scandinavian Mts. to the Ural Mts. in northeastern Europe and from the Balkans to the Alps, Sudetes and Carpathians (Caudullo et al. 2016). Additionally, the compact range of *P. mariana*, the species native to North America, extends from the mountains of Virginia in the south to the tundra in Alaska and Canada (Viereck et al. 1990). Considering the lack of strict specialization to specific soil conditions, a wide distribution of its host plants and localities with suitable climatic conditions, it is very likely that the distribution range of *T. wenchuanense* is much wider than currently known.

## Supplementary Information

Below is the link to the electronic supplementary material.

Supplementary file1 (TIFF 1251 KB)

Supplementary file2 (TIFF 3126 KB)

Supplementary file3 (TIFF 2188 KB)

Supplementary file4 (TIFF 2329 KB)

Supplementary file5 (TIFF 1173 KB)

Supplementary file6 (TIFF 2751 KB)

Supplementary file7 (DOCX 14 KB)

## Data Availability

The sequences presented in this article are available in the GenBank database under the indicated accession numbers. Fungal and mycorrhizal specimens (according to their herbarium vouchers) from the corresponding collections preserved in the herbaria of the Jagiellonian University in Kraków (KRA) or the University of L'Aquila (AQUI). The authors may be contacted for access.
