# Supplementary figures and images for: Tuber wenchuanense, a holarctic truffle with a wide range of host plants and description of its ectomycorrhiza with spruce
Source: Mycorrhiza. 2023 Jan 13;33(1-2):45–58. doi: 10.1007/s00572-022-01097-y (PMC9938020; doi:10.1007/s00572-022-01097-y)

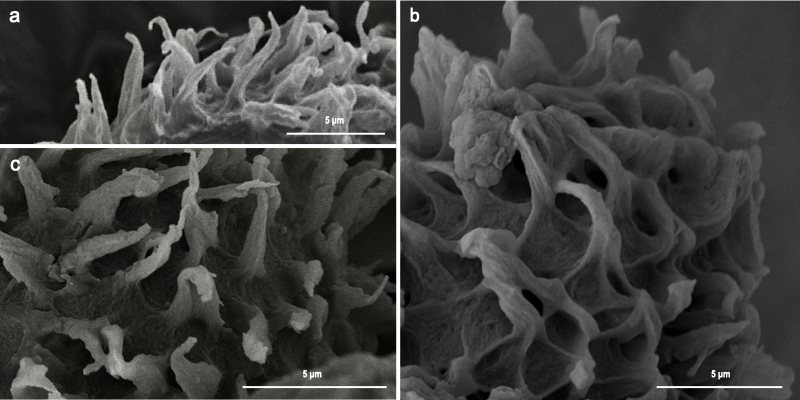

Supplement: Supplementary file 1 — Supplementary file1 (TIFF 1251 KB) [file 572_2022_1097_MOESM1_ESM.tiff]

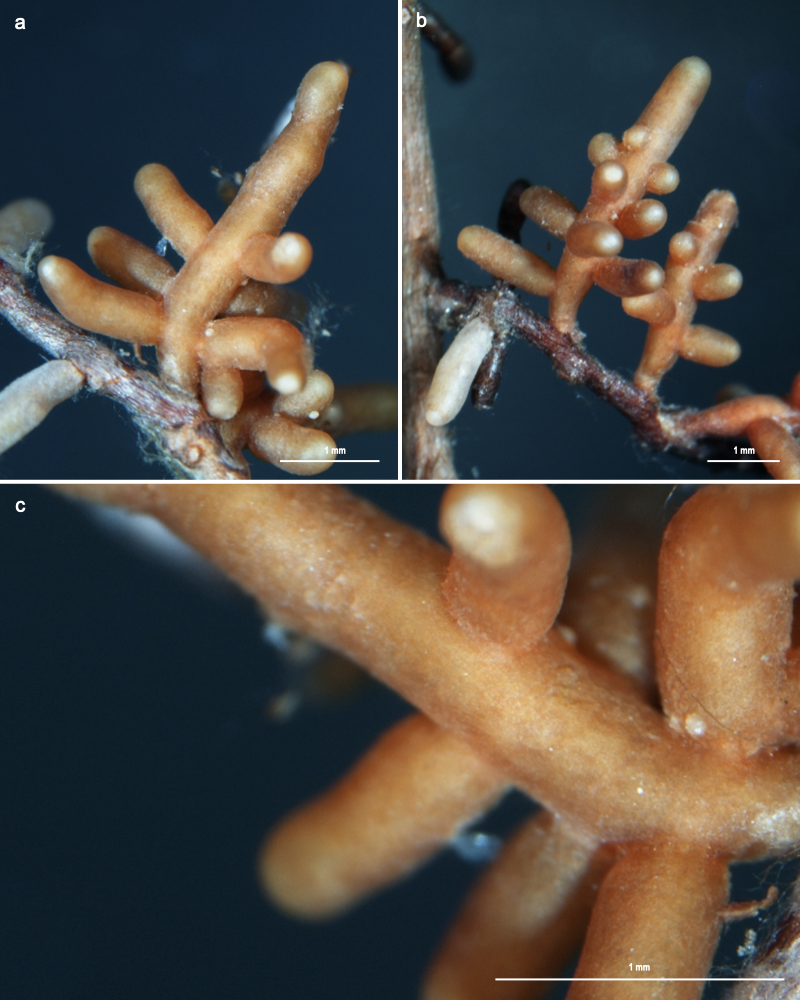

Supplement: Supplementary file 2 — Supplementary file2 (TIFF 3126 KB) [file 572_2022_1097_MOESM2_ESM.tiff]

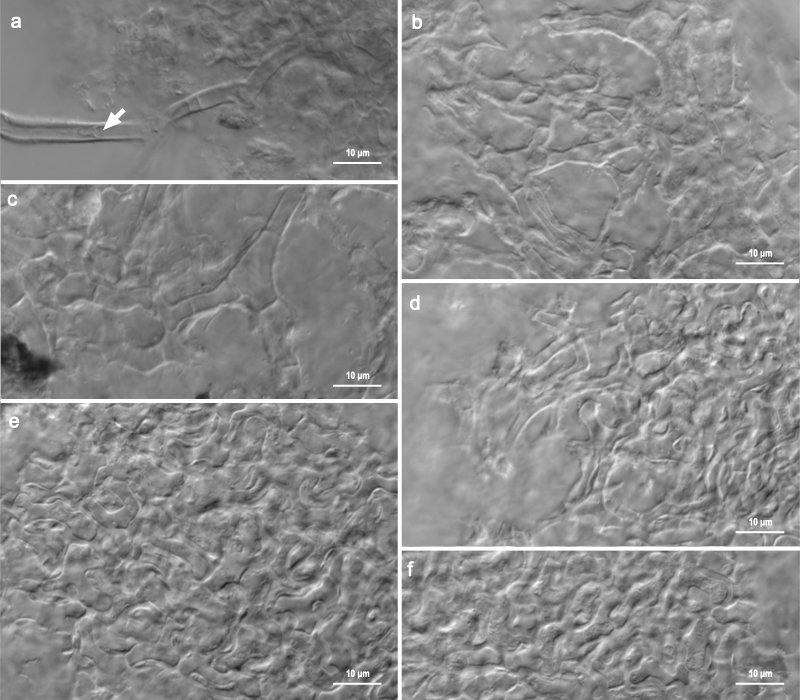

Supplement: Supplementary file 3 — Supplementary file3 (TIFF 2188 KB) [file 572_2022_1097_MOESM3_ESM.tiff]

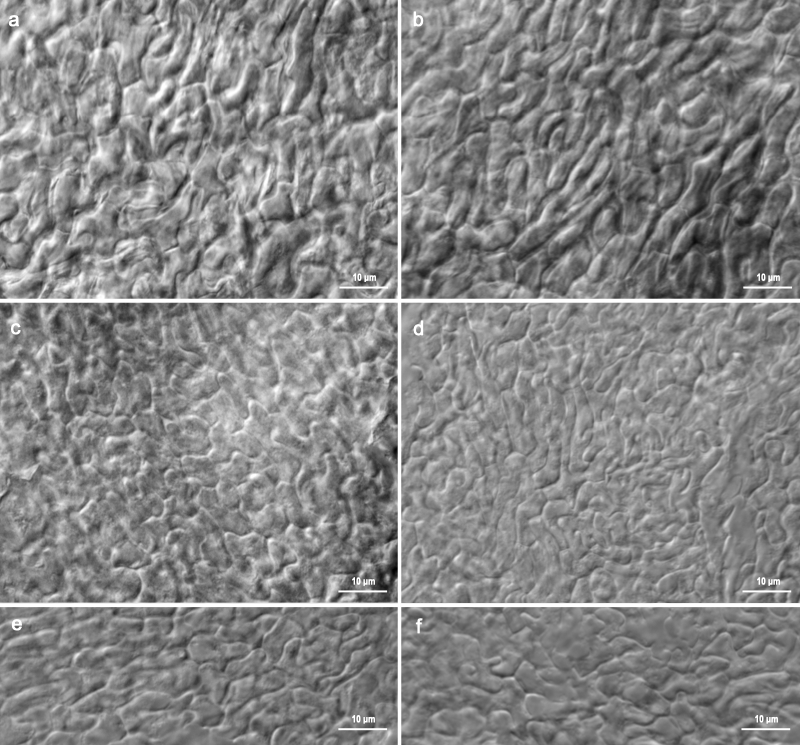

Supplement: Supplementary file 4 — Supplementary file4 (TIFF 2329 KB) [file 572_2022_1097_MOESM4_ESM.tiff]

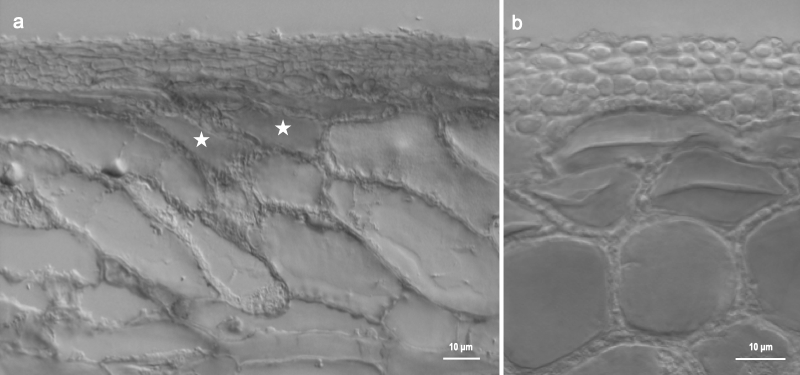

Supplement: Supplementary file 5 — Supplementary file5 (TIFF 1173 KB) [file 572_2022_1097_MOESM5_ESM.tiff]

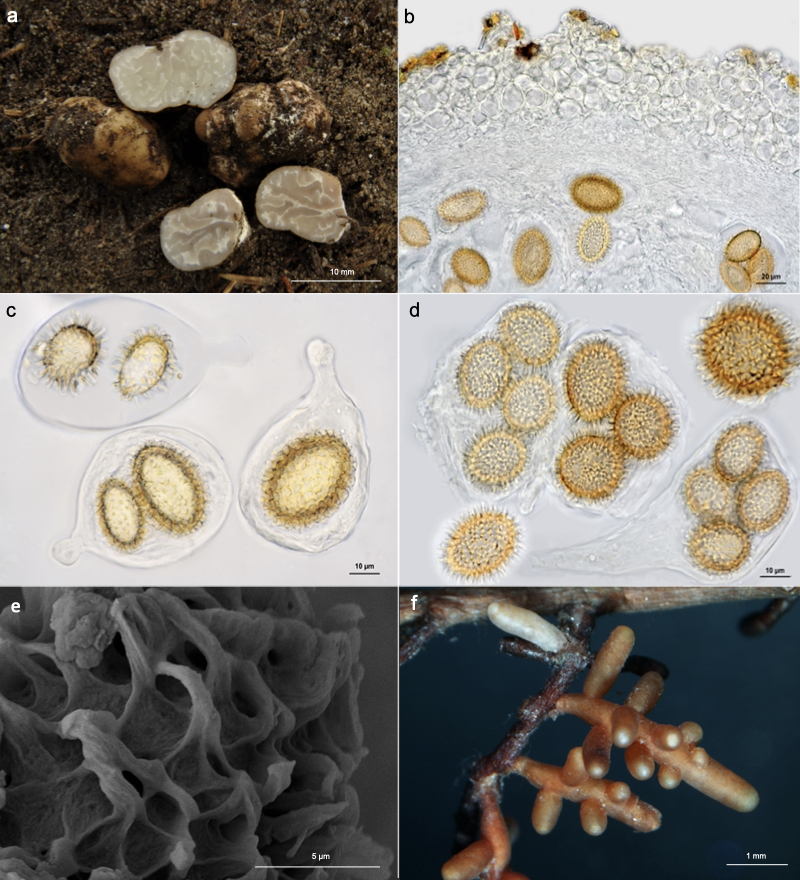

Supplement: Supplementary file 6 — Supplementary file6 (TIFF 2751 KB) [file 572_2022_1097_MOESM6_ESM.tiff]
