# Supplementary material for: Tuber wenchuanense, a holarctic truffle with a wide range of host plants and description of its ectomycorrhiza with spruce
Source: Mycorrhiza. 2023 Jan 13;33(1-2):45–58. doi: 10.1007/s00572-022-01097-y (PMC9938020; doi:10.1007/s00572-022-01097-y)
Supplement: Supplementary file 7 — Supplementary file7 (DOCX 14 KB) [file 572_2022_1097_MOESM7_ESM.docx]

Ascoma morphology

*Tuber wenchuanense* L. Fan & J.Z. Cao, in Fan, L., Cao, J.Z., Hou, C.L., Mycotaxon 123: 99, (2013), MycoBank MB#800577.

Ascoma 8-15 mm globose or slightly lobed; surface yellowish (R from Pale Luteous 11 to ochraceous 44, honey 64), smooth sometimes with rare prominent round warts, (Fig. S-6a); gleba immature whitish, mature light hazelnut brown (R. buff 45 to rosy buff 64) with scarce whitish venae externae: smell rather unpleasant, resembling radish or car grease.

Peridium 80–290 μm thick, pseudoparenchymatous with textura globulosa-angularis formed by subglobose, irregular or polygonal cells 7–30 μm in diameter, walls up to 4 μm thick, collenchymatic type, with the larger cells in the central part mixed with smaller cells, slightly yellowish; inner part 60–140 μm thick, prosenchymatous with the cells 2–5 μm wide, hyaline, thin-walled, well distinguished from the outer layer with slightly intertwined hyphae with major axis oriented parallel to the surface (periclineal) which in the innermost part extend into the sterile veins (Fig. S-6b).

Gleba with scarce sterile and ramified sterile veins of white colour, hyphae hyaline, 2–8 μm wide, with fasciculated parallel trend.

Asci hyaline 80–90 × 50–90 μm, ellipsoid to subglobose or pyriform, containing 1–5 spores, at the beginning with a stocky and robust stalk (20–22 × 9–12 μm) that tends to shrink when ripe, with a crozier to the foot.

Ascospores 20–41 × 16–37 μm, on average 29.7 ± 5.60 × 24.6 ± 4.41 μm, Q 1.00–1.54, on average 1.24 ± 0.13, excluding ornamentation, yellow (R. 12 luteous to amber 47), globose to ellipsoidal, frequently broadly ellipsoidal, often apiculate; highly variable ornamentation 3.5–10 μm with spines long straight or flexuous, especially in the early stages of maturation, after with the spine tips folded into a hook or spino-reticulated (Fig. S-6e), typically with 8–14 meshes across the ascospore width (Fig. S-6 c-d); spore walls to 3 μm thick. Endosporium generally clearly visible.

Ascospores from 1-spored asci 32–41 × 28–34 μm, 2-spored asci 26–40 × 20–28 μm, 3-spored asci 22–34 ×20–26 μm, 4-spored asci 20–34 × 16–26 μm, 5-spored asci (infrequent) 26–28 × 22–25 μm.

Specimens examined: POLAND, Tatra National Park, Western Tatra Mts., Western Carpathians, Za Bramką valley, 15.08.2019, *Picea abies* and *Fagus sylvatica* (*Dentario glandulosae-Fagetum* plant association), 910 m a.s.l., leg. M. Kozak, F. Karpowicz (KRA F-TPN/19/0182); at the junction of the Waksmundzki stream and the Białka stream, 21.09.2019, in the forest with *Picea abies* and *Alnus incana* (*Alnetum incanae* plant association), 1000 m a.s.l., leg. M. Kozak, F. Karpowicz (KRA F-TPN/19/0283 & AQUI 10349; 19/0290 & AQUI 10352;19/0293 & AQUI 10348; 19/0298 & AQUI 10350); ibidem, 03.10.2020, leg. M. Kozak, F. Karpowicz, J. Brańka (KRA F-TPN/20/0001 & AQUI 10347; 20/0003 & AQUI 10346; 20/0004 & AQUI 10353).
